# Scenario Projections of Respiratory Syncytial Virus Hospitalizations Averted Due to New Immunizations

**DOI:** 10.1001/jamanetworkopen.2025.14622

**Published:** 2025-06-11

**Authors:** Chelsea L. Hansen, Lawrence Lee, Samantha J. Bents, Amanda C. Perofsky, Kaiyuan Sun, Lea M. Starita, Amanda Adler, Janet A. Englund, Eric J. Chow, Helen Y. Chu, Cécile Viboud

**Affiliations:** 1 Brotman Baty Institute, University of Washington, Seattle; 2 Fogarty International Center, National Institutes of Health, Bethesda, Maryland; 3 PandemiX Center of Excellence, Roskilde University, Roskilde, Denmark; 4 Public Health-Seattle & King County, Seattle, Washington; 5 Emmett Interdisciplinary Program in Environment and Resources, Stanford University, Stanford, CA; 6 Department of Genome Sciences, University of Washington, Seattle; 7 Seattle Children’s Research Institute, Seattle, Washington; 8 Division of Allergy and Infectious Diseases, Department of Medicine, University of Washington, Seattle; 9 Department of Epidemiology, School of Public Health, University of Washington, Seattle

## Abstract

**Importance:**

In 2023, new immunization strategies became available for preventing respiratory syncytial virus (RSV) hospitalizations in infants and older adults. Modeling studies to understand the population-level impact of their use are important for public health planning.

**Objective:**

To estimate the number of hospitalizations averted in 2023 to 2024 due to new RSV immunization strategies and provide scenario projections for future seasons.

**Design, Setting, and Participants:**

This decision analytical model examined RSV hospitalizations in King County, Washington, from October 7, 2023, through April 26, 2025. The population of King County was disaggregated into infants younger than 6 months, infants aged 6 to 11 months, children aged 1 to 4 years, children/adults aged 5 to 59 years, adults aged 60 to 74 years, and adults aged 75 years or older.

**Exposures:**

Respiratory syncytial virus vaccination for adults aged 60 years or older, maternal RSV vaccination, and long-acting monoclonal antibodies (nirsevimab) for infants younger than 8 months.

**Main Outcomes and Measures:**

The proportion of RSV hospitalizations averted in adults aged 60 years or older and infants younger than 1 year were estimated using an RSV transmission model calibrated to RSV hospitalizations.

**Results:**

The RSV transmission model simulated the population of King County, which includes approximately 2.3 million individuals, with 23 700 infants younger than 1 year and 446 500 adults aged 60 years or older. During the 2023 to 2024 RSV season, 21.2% of adults aged 60 to 74 years, 32.7% of adults aged 75 years or older, and 33.0% of infants were protected through active or passive immunization. A total of 125 (95% projection interval [PI], 77-192) RSV hospitalizations were averted, with most of the benefit observed in infants younger than 6 months (28.6% [95% PI, 26.9%-30.5%] reduction from baseline) and adults aged 75 years or older (14.8% [95% PI, 14.3%-15.5%] reduction from baseline). For the 2024 to 2025 season, optimistic scenarios of high immunization coverage (50% in older adults and 80% in infants) projected reductions of 29.8% (95% PI, 29.1%-30.8%) in adults aged 75 years or older and 68.8% (95% PI, 66.0%-71.7%) in infants younger than 6 months compared with a counterfactual scenario with no immunizations. Targeting infants eligible for catch-up doses of nirsevimab early in the season increased the proportion of RSV hospitalizations averted in infants aged 6 to 11 months from 31.7% (95% PI, 29.4%-33.9%) to 40.4% (95% PI, 39.0%-42.1%). If vaccine protection in adults aged 75 years or older waned by 50% in the second year after immunization, the proportion of RSV hospitalizations averted was projected to decrease to 22.2% (95% PI, 21.7%-23.0%).

**Conclusions and Relevance:**

In this decision analytical model of RSV immunizations, the results suggest a modest reduction in RSV-diagnosed hospitalizations during the 2023 to 2024 season due to limited availability of immunization products, particularly for infants. A higher uptake earlier in the season may lead to substantial reductions in RSV hospitalizations in the 2024 to 2025 season.

## Key Points

**Question:**

How many respiratory syncytial virus (RSV)-diagnosed hospitalizations were averted in King County, Washington, during the 2023 to 2024 season due to new active and passive RSV immunization strategies, and how can disease reduction strategies be optimized in future seasons?

**Findings:**

In this analytical model study simulating a population of approximately 2.3 million individuals, moderate reductions in RSV hospitalizations during the 2023 to 2024 season were associated with modest coverage. With higher levels of coverage achieved early in the season, 68.8% of RSV hospitalizations in infants younger than 6 months and 29.8% in adults aged 75 years or older were projected to be avoided in the 2024 to 2025 season.

**Meaning:**

These findings suggest that RSV immunizations may be a powerful tool for preventing hospitalizations and that modeling studies may support public health strategies to optimize immunization coverage.

## Teaser

This decision analytical model study estimates the number of respiratory syncytial virus (RSV) hospitalizations averted due to new RSV immunization strategies in King County, Washington, and provides scenario projections for future seasons.

## Introduction

Respiratory syncytial virus (RSV) is a leading cause of pediatric disease burden, with 1500 to 2800 hospitalizations per 100 000 infants younger than 1 year annually in the US.^1,2,3^ Respiratory syncytial virus is also responsible for substantial disease burden in older adults (aged ≥75 years), though testing is less frequent and less sensitive in this age group.^1,4^ Prior to 2023, there were no RSV vaccines for adults, and the only available prophylaxis against severe RSV disease in infants was palivizumab, which was reserved for very premature newborns and infants with high-risk conditions.^5,6^ In 2023, a new long-acting monoclonal antibody, nirsevimab, was recommended for routine use in infants along with 2 vaccines for older adults, one of which was also recommended for use in pregnant women to protect newborns.^7,8,9^

Observational studies have evaluated the outcomes associated with these immunizations over the course of the 2023 to 2024 season,^10,11,12,13,14,15^ but modeling studies to understand the population-level impact of their use and guide public health planning remain scarce. Historically, there have been many modeling efforts to deliver short-term forecasts and scenario projections for influenza and COVID-19, helping to guide the public health response to these pathogens.^16,17,18^ In anticipation of new immunization products, there has been growing interest in expanding this work to include RSV,^19,20,21,22,23,24^ but modeling efforts remain limited, especially at the local scale in which decision making and planning are done. In this study, we leveraged routinely collected county public health surveillance data in King County, Washington, to develop an RSV transmission model and project the number of RSV-diagnosed hospitalizations averted in King County during the 2023 to 2024 RSV season due to new RSV immunization strategies. We provide scenario projections and implications for future RSV seasons.

## Methods

### RSV Hospitalization and Demographic Data

This decision analytical model focused on Washington’s King County, which is the state’s most populous county with approximately 2.3 million individuals and 19 hospitals. As the study involved non–human participants research, the University of Washington Institutional Review Board deemed it exempt from approval and informed consent. The study followed the Consolidated Health Economic Evaluation Reporting Standards (CHEERS) reporting guidelines for decision analytical models.

More than 80% of all pediatric hospitalizations, including those with diagnosed RSV, occur at Seattle Children’s Hospital, the region’s largest pediatric hospital. For children younger than 10 years, we used hospitalizations at Seattle Children’s Hospital with positive RSV test results (during or preceding admission) from July 1, 2017, through April 27, 2024. For individuals older than 10 years, we used RSV-diagnosed hospitalizations among King County residents or at King County hospitals from Washington State’s syndromic surveillance platform for the same period (eMethods in Supplement 1).^25,26^ Data were aggregated into a weekly all-ages time series and summarized by season (defined as the first week of July to the last week of June the following year) and age group (<6 months, 6-11 months, 1-4 years, 5-59 years, 60-74 years, and ≥75 years). Values between 1 and 9 in the weekly time series had been suppressed for privacy issues and were imputed for subsequent modeling using linear interpolation. We rescaled weekly hospitalizations prior to April 2020 to reflect changes in RSV testing and reporting during the COVID-19 pandemic (eMethods and eFigure 1 in Supplement 1). We extracted age-specific population sizes, birth rates, and net migration rates for King County using the tidycensus package in R.^27^ We used a Washington-specific contact matrix described by Mistry et al^28^ to define contacts within and between age classes.

### Immunization Data

We obtained monthly data on RSV immunizations for King County from July 1, 2023, through April 30, 2024, from the Washington State Immunization Information System.^29^ Data included the doses of nirsevimab for infants younger than 1 month to 7 months and RSV vaccines for adults aged 60 to 74 years and 75 years or older. The Washington State Immunization Information System did not have data based on pregnancy status, so we assumed that vaccines administered to female residents between age 15 and 49 years were related to pregnancy. We assumed a 1-month lag between vaccine administration during pregnancy and infant protection after birth (ie, doses administered to pregnant women in September provide protection to infants born in October). We refer to doses of nirsevimab administered to eligible infants born prior to October 1, 2023, as catch-up doses and nirsevimab doses administered to infants born during the RSV season as birth doses. Additional details on infant immunization coverage are provided in the eMethods and eTable 1 in Supplement 1.

### Statistical Analysis

All analyses were performed using R, version 4.3.1 (R Foundation for Statistical Computing). We developed an age-structured compartmental RSV transmission model adapted from Pitzer et al.^30^ The model assumes short-term maternally derived partial immunity, repeated infections throughout life, a short period of sterilizing immunity following infection, and a gradual buildup of permanent partial immunity. The model diagram and parameter values are provided in the eMethods, eTable 2, and eFigures 2 and 3 in Supplement 1. We used maximum likelihood estimation to calibrate the model to the RSV hospitalization data prior to October 1, 2023. Parameters for susceptibility and infectiousness were fixed based on published literature,^20,22,30^ while parameters for seasonality, reporting rates, and changes in contact patterns during COVID-19 were fitted to King County data (eMethods, eTable 2, and eFigure 4 in Supplement 1). We used the maximum likelihood approach to generate bounds for our fitted parameters (eMethods in Supplement 1) and Latin Hypercube Cube sampling to obtain 100 model trajectories consistent with the data from these bounds. We report the median and 2.5% and 97.5% quantiles from these 100 trajectories as 95% projection intervals (PIs).

After fitting the data up to October 1, 2023, we let the 100 model trajectories project forward until April 27, 2024. These projections represent our counterfactual scenario (no immunizations) for the 2023 to 2024 season. We next ran projections for the 2023 to 2024 season using the reported immunization coverage. We used data from clinical trials and observational studies to set the effectiveness of immunizations against hospitalization in our transmission model (eMethods, eTable 2, and eFigure 5 in Supplement 1).^10,11,12,13,14,31,32,33,34^ We subtracted these immunization-based projections from our counterfactual to arrive at an estimated number of RSV-diagnosed hospitalizations averted due to immunizations for the 2023 to 2024 season.

#### Scenarios for the 2024 to 2025 Season

For the 2024 to 2025 season, we established 2 dimensions of immunization coverage (optimistic and pessimistic) for older adults and infants, resulting in 4 intervention scenarios plus a counterfactual scenario (Table 1).^35,36,37,38^ Following the vaccination guidance for the 2024 to 2025 season,^39^ we assumed that older adults who were vaccinated in the 2023 to 2024 season would not get revaccinated in the 2024 to 2025 season, as vaccine protection is expected to last for at least 2 years. In the counterfactual scenario, we assumed that there were no immunizations for infants or adults aged 60 years or older in either the 2023 to 2024 or 2024 to 2025 seasons. In the pessimistic scenarios, we assumed that the cumulative coverage in adults aged 60 years or older was 37.5% (including the 2023-2024 coverage) and that the combined coverage of nirsevimab and maternal vaccination was 40% in infants. In the optimistic scenarios, we assumed 50% coverage in older adults and 80% coverage in infants (additional details provided in the Results).

**Table 1.** Scenarios for the 2024 to 2025 RSV Season^a^

Long-lasting monoclonal antibodies (nirsevimab) for infants aged <8 mo (assuming an average effectiveness of 80% over 6 mo) and maternal vaccination (assuming an average effectiveness of 57% over 6 mo)	Vaccination of adults aged ≥60 y, assuming an average effectiveness of 75% for 2 y		
	Optimistic coverage, 50% for adults aged ≥75 y (67% coverage) and 60-74 y (43% coverage)^b^	Pessimistic coverage, 37.5% for adults aged ≥75 y (50% coverage) and 60-74 y (32% coverage)^b^	Counterfactual, no coverage in 2024-2025 or 2023-2024
Optimistic coverage, 80%^c^; catch-up coverage, 74%^d^; birth coverage, 87% (nirsevimab, 47%; maternal vaccination, 40%)^e^	Scenario A	Scenario B	NA
Pessimistic coverage, 40%; catch-up coverage, 25%^d^; birth coverage, 58% (nirsevimab, 31%; maternal vaccination, 27%)^e^	Scenario C	Scenario D	NA
Counterfactual, no coverage in the 2024-2025 season; coverage in the 2023-2024 season does not impact this season	NA	NA	Scenario E

Abbreviations: NA, not applicable; RSV, respiratory syncytial virus.

^a^ Parameters for immunization effectiveness and duration of protection were fixed across scenarios. There were 2 dimensions of coverage (optimistic and pessimistic) for older adults and infants, resulting in 4 scenarios plus a counterfactual scenario. Coverage levels were based on the observed data in the 2023 to 2024 season, with upper bounds based on coverage for other immunizations.

^b^ Coverage included the observed coverage from the 2023 to 2024 season (25%). In the optimistic scenario, coverage is doubled, and in the pessimistic scenario, coverage is increased by 50%. Optimistic coverage in adults aged 60 to 74 years should not exceed 50% (assuming that 50% of this population is eligible due to a high-risk condition).^36^ Optimistic coverage for adults aged 75 years or older does not exceed influenza vaccine coverage in adults aged 65 years or older in Washington State.^37^

^c^ Optimistic coverage for infants (80%) is based on hepatitis B birth dose coverage in Washington State.^35^

^d^ There were approximately 13 800 infants born before October 1, 2024, who were eligible for catch-up doses of nirsevimab. Pessimistic coverage for 2024 approximately doubles the observed coverage in the 2023 to 2024 season (more details provided in eTable 1 in Supplement 1).

^e^ There were approximately 11 900 infants born between October 1, 2024, and March 31, 2025. These infants were either born to vaccinated mothers or were eligible for a birth dose of nirsevimab. Pessimistic coverage for the 2024 to 2025 season is approximately the same as the observed coverage in the 2023 to 2024 season (more details provided in eTable 1 in Supplement 1).

#### Sensitivity Analysis

In sensitivity analysis (using the most optimistic scenario [scenario A in Table 1]), we examined alternative strategies for infant immunization. In scenario A2, the total coverage remained at 80%, but we extended the duration of protection for nirsevimab from 180 days to 270 days. In scenario A3, all catch-up doses of nirsevimab were administered from October to November 2024. In scenario A4, all protection was given via nirsevimab (no maternal vaccination), while in scenario A5, we assumed that all newborns were protected by maternal vaccination (no nirsevimab birth doses). In the main analysis for adults aged 60 years or older, we assumed that the vaccine remained effective for 2 years and that vaccine protection was against hospitalization given infection but did not prevent infection. In sensitivity analysis, we considered vaccine waning, with protection reduced by 50% in the second year after administration. We also considered that vaccination reduced the risk of infection. The eMethods in Supplement 1 provide more detail on these sensitivity analyses.

## Results

### RSV Epidemics in King County

**Figure 1.**
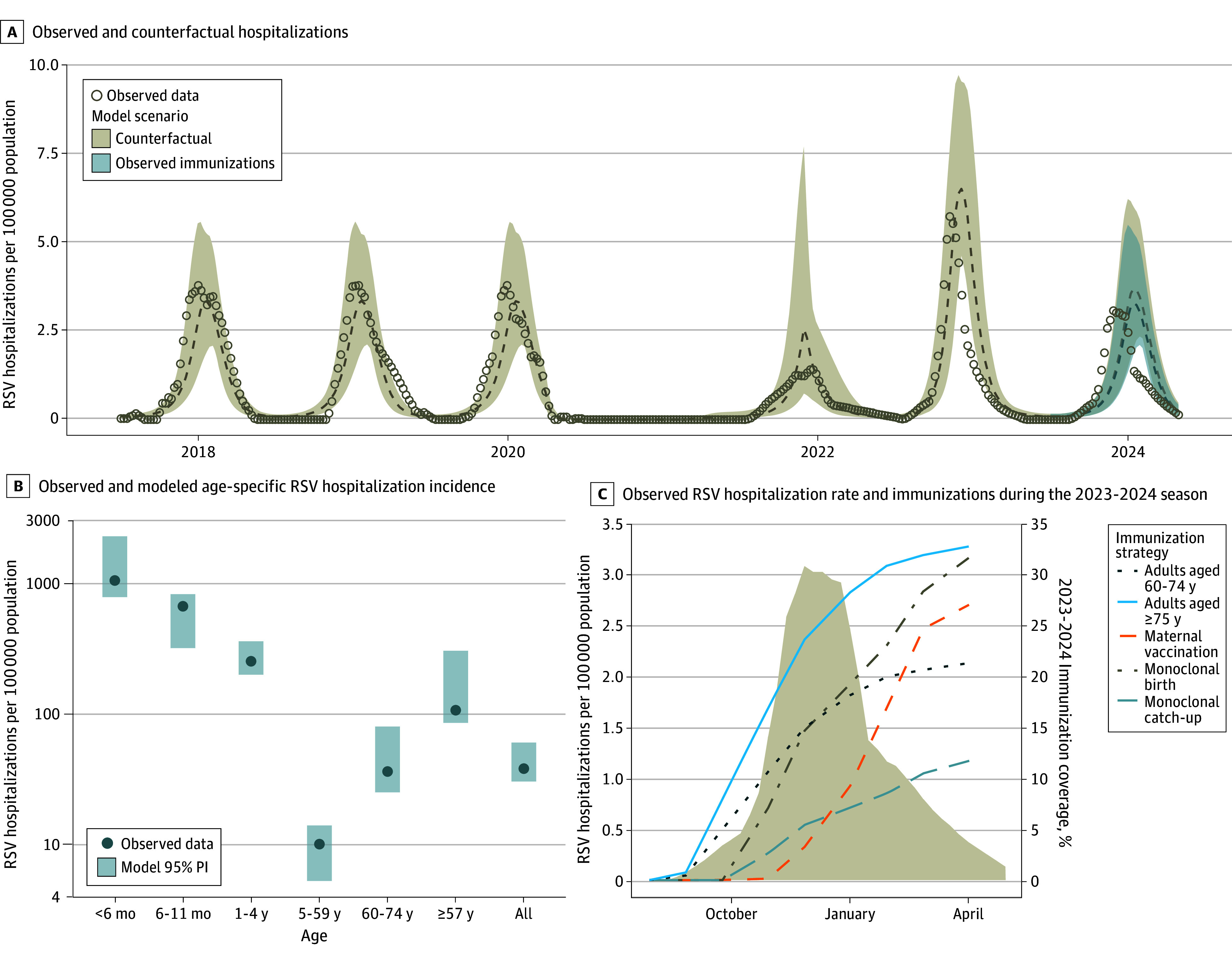
Reported Respiratory Syncytial Virus (RSV) Hospitalizations, Immunizations, and Model Projections in King County, Washington, 2023 to 2024 Season A, Observed RSV hospitalizations are after adjusting for RSV testing (eMethods in Supplement 1). Dashed line indicates median; shaded areas, 95% projection intervals (PIs).

The RSV transmission model simulated the population of King County of approximately 2.3 million individuals, including 23 700 infants younger than 1 year and 446 500 adults aged 60 years or older. Prior to the COVID-19 pandemic, RSV in King County followed a winter seasonal pattern, with peaks typically occurring in January (Figure 1A). Due to public health interventions in response to the COVID-19 pandemic, there was minimal circulation of RSV during the 2020 to 2021 winter season. As COVID-19 restrictions eased, RSV activity resumed in the summer of 2021, followed by a large peak in November 2022. The timing of the 2023 to 2024 RSV season was closer to that of prepandemic seasons, with a peak in early December 2023. The model accurately reproduced the incidence and age distribution of RSV hospitalizations throughout 2018 to 2024 but projected a slightly later season in 2023 to 2024 than observed (Figure 1A and B).

### RSV Immunizations and Hospitalizations During the 2023 to 2024 Winter Season

By the end of March 2024, 21.2% of adults aged 60 to 74 years and 32.7% of adults aged 75 years or older had received an RSV vaccine (Figure 1C). Among infants born between October 2023 and March 2024, 27.1% were born to vaccinated mothers, and an additional 31.1% received a birth dose of nirsevimab, resulting in an overall coverage of 58.2% for newborns. For infants younger than 8 months at the start of the RSV season, 11.4% received a catch-up dose of nirsevimab.^40^ Overall, 33.0% of all eligible infants were protected by immunization (eTable 1 in Supplement 1). However, the effective coverage at the time of the RSV peak in early December was 18.5% for older adults and 21.7% for infants.

In observed hospitalization data for the 2023 to 2024 RSV season, the RSV hospitalization rate was 38.9 per 100 000 population. Our counterfactual model (assuming no immunizations) projected an RSV hospitalization rate per 100 000 population of 49.5 (95% PI, 34.5-69.8), while our immunization model (replicating the observed coverage in this season) projected 44.0 (95% PI, 31.0-61.4) RSV hospitalizations per 100 000 population. This projection amounts to a 10.9% (95% PI, 8.8%-13.0%) reduction (Table 2) or 125 (95% PI, 77-192) RSV hospitalizations averted. The reductions were greater in the age groups with the highest immunization coverage, including a 14.8% (95% PI, 14.3%-15.5%) reduction in adults aged 75 years or older and a 28.6% (95% PI, 26.9%-30.5%) reduction in infants younger than 6 months. There was no difference among scenarios in the age groups of 1 to 4 years and 5 to 59 years, which were not targeted by immunizations. For each RSV hospitalization averted, there were 4494 (95% PI, 2377-7635) vaccines administered in adults aged 60 to 74 years and 1188 (95% PI, 602-2171) vaccines administered in adults aged 75 years or older. In infants, 103 (95% PI, 63-181) doses of nirsevimab and 177 (95% PI, 105-290) maternal vaccinations were administered for each RSV hospitalization averted.

**Table 2.** Modeled Scenario for the 2023 to 2024 Season Based on Observed Immunization Coverage and Scenario Projections for the 2024 to 2025 RSV Season

RSV hospitalizations averted	Projections				
	2023-2024 Season	2024-2025 Season^a^			
	Modeled	Scenario A	Scenario B	Scenario C	Scenario D
**By age group, per 100 000 (95% PI)**					
<6 mo	563.3 (323.2-921.5)	1322.6 (749.3-2186.0)	1322.8 (749.2-2185.8)	670.3 (376.3-1100.8)	670.2 (376.3-1100.7)
6-11 mo	29.0 (18.9-45.3)	175.1 (106.2-271.9)	175.1 (106.2-271.8)	64.7 (39.4-100.1)	64.7 (39.4-100.1)
60-74 y	4.7 (2.8-8.9)	9.5 (5.3-17.3)	7.1 (4.0-13.1)	9.5 (5.3-17.3)	7.1 (4.0-13.1)
≥75 y	27.5 (15.0-54.3)	55.7 (30.4-105.5)	42.1 (23.0-80.0)	55.7 (30.4-105.5)	42.1 (23.0-80.0)
All ages	5.5 (3.3-8.7)	12.6 (8.0-20.5)	11.7 (7.2-18.7)	8.4 (5.4-13.8)	7.4 (4.7-12.0)
**By age group, % (95% PI)**					
<6 mo	28.6 (26.9-30.5)	68.8 (66.0-71.7)	68.8 (66.0-71.7)	34.7 (33.0-36.6)	34.7 (33.0-36.6)
6-11 mo	5.2 (4.0-6.4)	31.7 (29.4-33.9)	31.7 (29.4-33.9)	11.7 (10.6-12.7)	11.7 (10.6-12.7)
60-74 y	9.7 (9.3-10.2)	19.5 (18.9-20.4)	14.7 (14.2-15.4)	19.5 (18.9-20.4)	14.7 (14.2-15.4)
≥75 y	14.8 (14.3-15.5)	29.8 (29.1-30.8)	22.5 (22.0-23.3)	29.8 (29.1-30.8)	22.5 (22.0-23.3)
All ages	10.9 (8.8-13.0)	26.2 (20.9-30.9)	24.0 (18.7-28.5)	17.8 (14.5-21.0)	15.4 (12.5-18.0)

Abbreviations: PI, projection interval; RSV, respiratory syncytial virus.

^a^ Scenarios described in Table 1.

### Scenario Projections for the 2024 to 2025 Season

**Figure 2.**
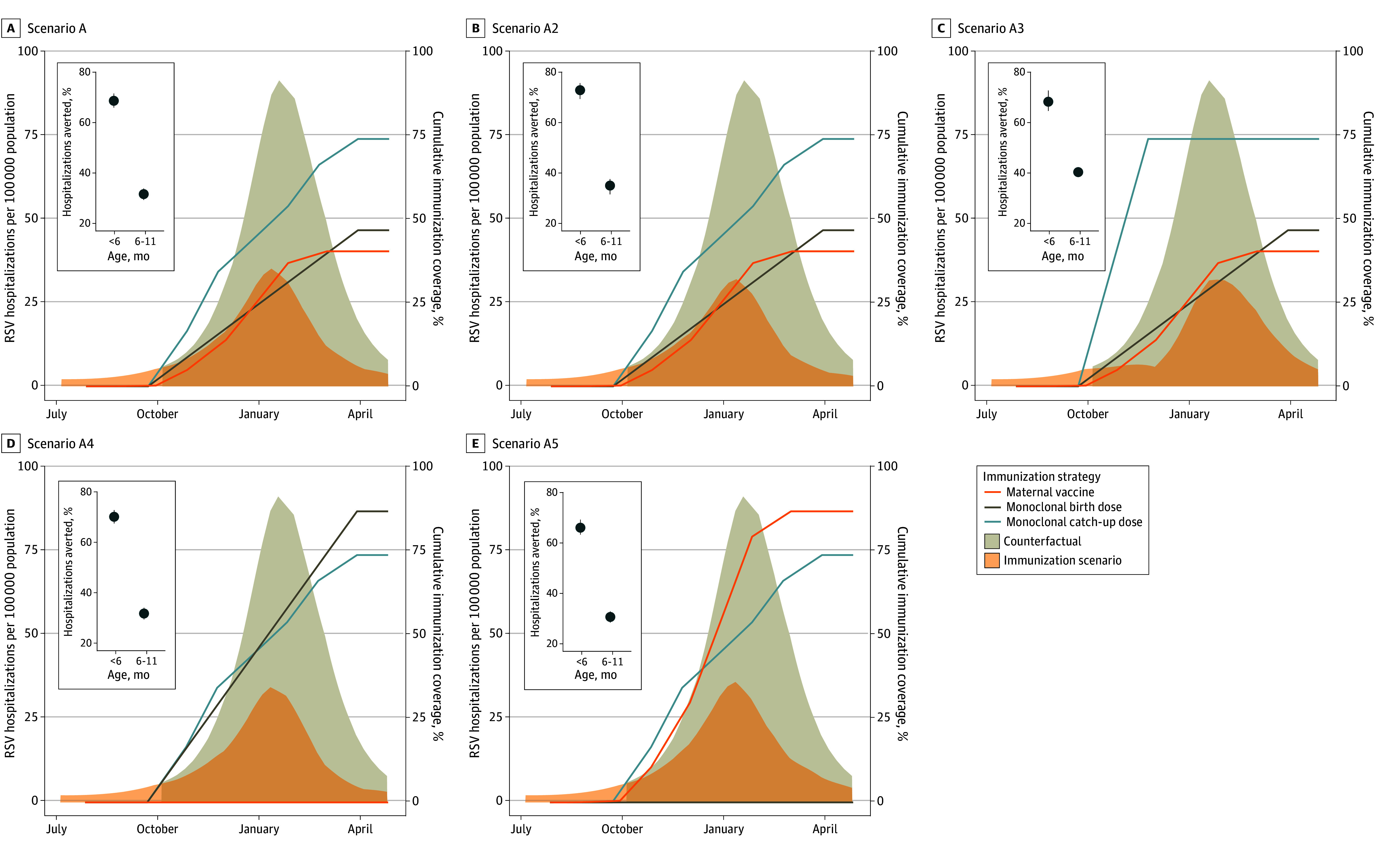
Sensitivity Analyses Exploring Alternative Scenarios for Infants in the 2024 to 2025 Respiratory Syncytial Virus (RSV) Season Using the optimistic scenario (scenario A in Table 1) for the 2024 to 2025 season, we explored alternative immunization strategies in infants, assuming that the same total number of infants were immunized (80% of eligible infants). In scenario A2, all conditions are the same as scenario A except that the duration of protection for monoclonal antibodies (nirsevimab) is extended from 180 to 270 days. In scenario A3, the total coverage is the same but all catch-up doses of nirsevimab are administered by the end of November. In scenario A4, all immunization coverage is achieved through nirsevimab (no maternal immunization). In scenario A5, the catch-up doses of nirsevimab stay the same, but all newborn immunization is achieved through maternal vaccination (no nirsevimab birth doses). The insets show the percent reduction under each immunization scenario for infants younger than 6 months and aged 6 to 11 months compared with the counterfactual scenario of no immunizations. Error bars indicate the 95% projection interval.

**Figure 3.**
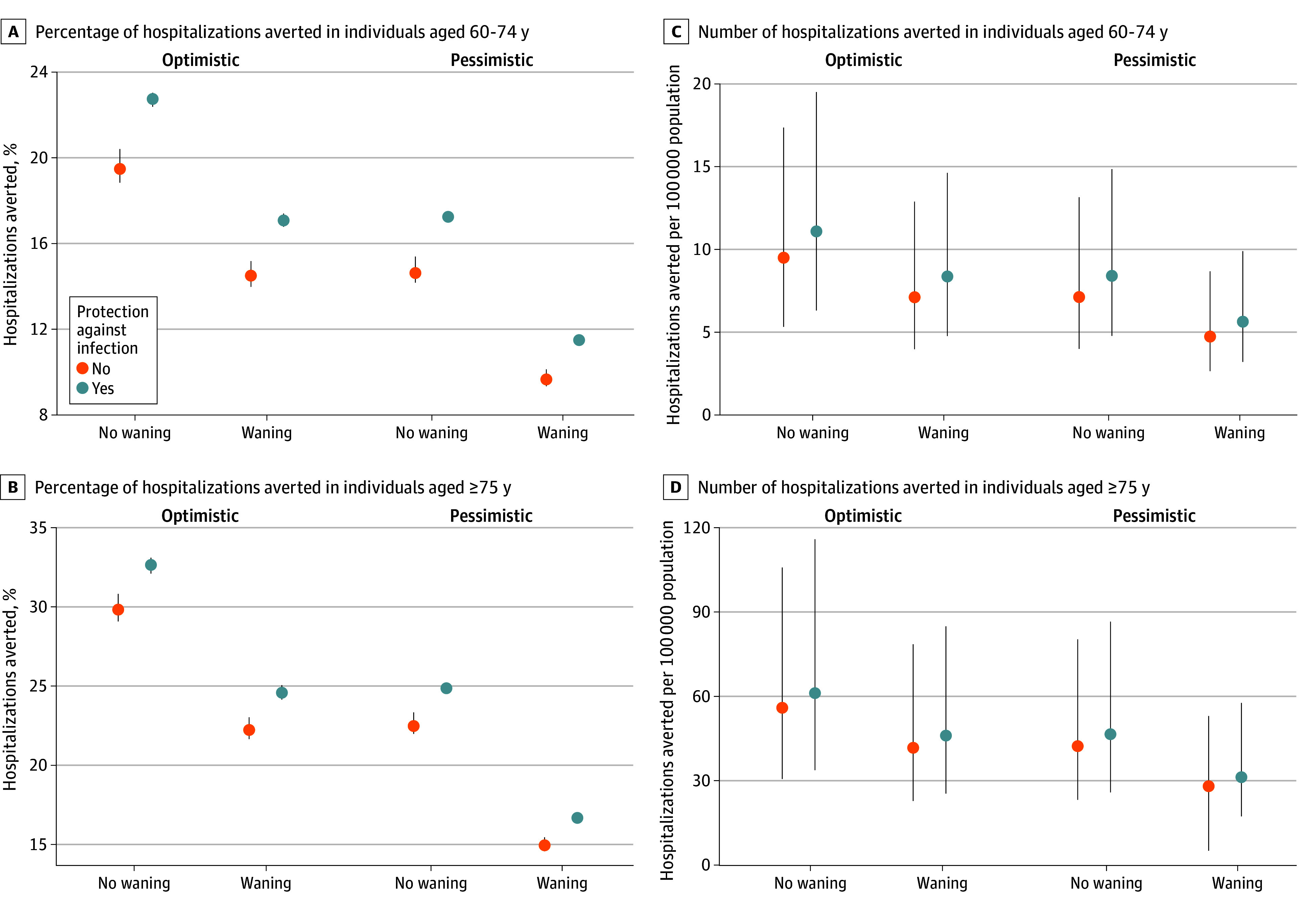
Sensitivity Analysis Exploring Alternative Scenarios for Older Adults in the 2024 to 2025 Respiratory Syncytial Virus (RSV) Season In our main analysis, we assumed that the vaccine protection in older adults was durable for 2 years and that the protection (assuming a vaccine effectiveness of 75%) was only against hospitalization given infection and did not protect against infection. In these sensitivity analyses, we considered how our estimates would change if protection from the vaccine was reduced by 50% in the second year and if the protection was a combination of protection against infection and hospitalization given infection (50% and 50% for a combined relative risk of 0.25; vaccine effectiveness, 75%). Error bars indicate 95% projection intervals.

Our most pessimistic scenario for the 2024 to 2025 season showed reductions in hospitalizations similar to the observed reductions in the 2023 to 2024 season. Our most optimistic scenario resulted in substantially greater reductions in RSV hospitalizations, including a 68.8% (95% PI, 66.0%-71.7%) reduction in infants younger than 6 months and a 29.8% (95% PI, 29.1%-30.8%) reduction in adults aged 75 years or older. Projected number of doses required for each hospitalization averted for the 2024 to 2025 season were 111 (95% CI, 97-134) for nirsevimab, 132 (95% PI, 113-159) for maternal vaccinations, 4460 (95% PI, 3669-5503) for adults aged 60 to 74 years, and 1173 (95% PI, 908-1399) for adults aged 75 years or older. In our sensitivity analysis testing the impact of different immunization strategies in infants, there were minimal differences between strategies for infants younger than 6 months (Figure 2). The greatest increase in immunization benefits occurred when all catch-up doses of nirsevimab were administered early in the season, but this mostly benefited infants aged 6 to 11 months (from 31.7% [95% PI, 29.4%-33.9%] to 40.4% [95% PI, 39.0%-42.1%] of RSV hospitalizations averted). In our sensitivity analysis testing waning assumptions in older adults, we found that if vaccine protection is reduced by 50% in the second year, the proportion of RSV hospitalizations averted in the optimistic scenario would decrease to 22.2% (95% PI, 21.7%-23.0%) among adults aged 75 years or older and from 19.5% (95% PI, 18.9%-20.4%) to 14.6% (95% PI, 14.0%-15.2%) among adults aged 60 to 74 years (Figure 3). If vaccines protect against infection, there is a marginal increase in both the direct benefits to older adults and indirect benefits to other age groups.

## Discussion

In this decision analytical model, we estimated the number of RSV hospitalizations averted during the 2023 to 2024 respiratory virus season in King County, Washington, associated with novel immunization products and produced scenario projections for the 2024 to 2025 season. By late March 2024, 25% of eligible older adults and 33% of infants were protected through RSV immunization, although a fraction of these immunizations were administered after peak RSV activity. We found measurable reductions in hospitalizations for the 2023 to 2024 season compared with the counterfactual scenario of no immunization (approximately 15% averted hospitalizations for adults aged ≥75 years and 29% for infants aged <6 months). In our optimistic scenario for the 2024 to 2025 season, we expect reductions of 30% and 70%, respectively.

During the first years of the COVID-19 pandemic, RSV circulation in King County^41^ and many other locations^42,43,44,45,46,47^ was disrupted. Multiple factors may have been associated with RSV circulation during the pandemic (eg, changing social contact patterns, waning immunity, delayed age at first infection), and as RSV circulation returned, the timing of the RSV season was uncertain. The 2023 to 2024 RSV season in King County was slightly earlier than prepandemic seasons. While our model projected a lingering effect of pandemic perturbations in 2023 to 2024, the modeled epidemic was not as early as observed. Irrespective of the mechanisms at play, an earlier 2023 to 2024 epidemic may have reduced the impact of immunizations administered late in the season. In addition, other factors that limited the benefits of immunizations are important to consider, including supply shortages for nirsevimab and confusion around vaccine eligibility for older adults.

Reporting for RSV immunizations is not mandatory, and there is uncertainty in the true level of immunization coverage during the 2023 to 2024 season, particularly for adults. Publicly available data from the National Immunization Survey have indicated that 31.9% of adults aged 60 years or older in Washington were vaccinated by the end of March 2024,^48^ which is slightly higher than our estimate of 25% for King County. We did not explicitly have data on vaccination among pregnant women, so we assumed doses administered to female residents aged 15 to 49 years were because of pregnancy. National data on coverage among pregnant persons indicate that 17.8% of eligible individuals were vaccinated by the end of January 2024,^49^ which is lower than our estimates for King County but relies on different methods for ascertaining coverage. We also did not have data on age at the time of nirsevimab administration. Publicly available Washington State Immunization Information System data on nirsevimab coverage have indicated that 11.4% of infants eligible for catch-up doses were immunized by March 2024.^40^ We assumed that the coverage in King County would be similar to this rate and treated the remaining nirsevimab doses as birth doses, resulting in 31.1% nirsevimab coverage for infants born during the RSV season. This rate is lower than that reported in National Immunization Survey data, which indicated 41.3% nirsevimab coverage among infants younger than 8 months in March 2024; however, the National Immunization Survey provides a cross-sectional coverage estimate that may not be directly comparable to the data we used.^50^

In addition to uncertainty regarding the true level of immunization coverage, several other model assumptions may have influenced our results, which we explored through sensitivity analyses. For older adults, we explored how the duration of protection and the assumed type of protection (ie, against infection vs against hospitalization without effect on infection) may change our results. We found that the duration of protection was more important than the type of protection in estimating the number of RSV hospitalizations averted. Likewise, the indirect benefits from reducing transmission in older adults were minimal in the general population. Current data have suggested that RSV vaccines for older adults may remain effective for 2 to 3 years.^51^ Accordingly, at the time of writing, it was only recommended that older adults receive a single dose of RSV vaccine.^39,52^ In May 2024, the Centers for Disease Control and Prevention updated its recommendations for RSV vaccination in older adults to target all adults aged 75 years or older and only those aged 60 to 74 years with high-risk conditions.^39^ While we did look at these age groups separately, we did not model high-risk subpopulations explicitly. We found greater vaccination benefits among adults aged 75 years or older, which may be partly due to higher vaccine coverage and the higher baseline risk of severe RSV disease in this age group as evidenced by the requirement of nearly 4 times as many vaccines to avert 1 hospitalization in adults aged 60 to 74 years vs those aged 75 years or older. Going forward, modeling studies may help inform both recommendations for revaccination among adults and the anticipated benefits of extending to additional age groups.

For infants, we considered how the duration of protection and timing of nirsevimab administration might impact our results. Assuming that nirsevimab does not provide any protection against infection,^53^ extending the duration of protection did not greatly increase the number of hospitalizations averted. However, this consideration may be more important in settings in which RSV has less pronounced seasonality. We saw the best outcomes in our scenario that administered nirsevimab catch-up doses early in the season, though this was largely confined to reductions in hospitalizations in older infants. For younger infants, we considered the tradeoff between maternal vaccination and nirsevimab birth doses. Assuming the same levels of coverage and a similar duration of protection, we found only a marginal difference in favor of nirsevimab (associated with our assumption of higher effectiveness for nirsevimab), suggesting that to maximize coverage in the infant population, leveraging all available immunization products may be a reasonable strategy.

### Limitations

Our study has a number of limitations to consider. We calibrated the model to RSV-diagnosed hospitalizations, which may have underestimated the true burden of RSV, particularly for adults. Additionally, testing for RSV has changed substantially since 2020. We attempted to adjust the pre-2020 data to better reflect current testing practices, but this may have influenced our projections for the 2023 to 2024 and 2024 to 2025 seasons. Of note, although within the projection intervals, our median estimate for the RSV hospitalization rate during the 2023 to 2024 season was slightly higher and peaked slightly later than the observed data, which may have inflated our estimates for the absolute number of hospitalizations averted. However, our model is still useful for understanding the relative differences between scenarios. Additionally, King County is home to many large, highly regarded health care facilities that serve patients from an extensive geographic area; thus, some of the RSV hospitalizations in our calibration data may have occurred in noncounty residents. Separate data from Washington’s syndromic surveillance platform indicate that 15% to 20% of emergency department visits in King County are from nonresidents; a similar proportion may apply to hospitalizations. Although the transmission model simulates the population of King County, we believed that including these noncounty residents in the calibration data and resulting scenario projections provided a more complete picture of the RSV burden on the King County hospital system, which has important implications for health care capacity planning during the respiratory virus season. Similarly, some of the immunization doses may have been administered to noncounty residents.

Finally, while it is important to extend modeling efforts to the local level, our sample sizes were small, and we did not have many historical years of hospitalization data for model calibration. We also had a fairly complex disease model necessary to capture the RSV postpandemic rebound and age structure, requiring a 2-step calibration process (eMethods in Supplement 1). Nevertheless, we were able to fit a transmission model using data sources typically available to local health departments and have provided an example of how partnerships between academic institutions and public health departments may increase modeling capacity at the local level and complement observational studies evaluating vaccine effectiveness.^10,11,12,13,14,15^

## Conclusions

This decision analytical model of RSV immunizations in King County, Washington, found that routinely collected public health surveillance data may inform transmission models for scenario projections at the local level. The results suggest a modest reduction in RSV hospitalizations during the 2023 to 2024 season. Scenario projections for the 2024 to 2025 season suggested that two-thirds of RSV hospitalizations could be averted in infants younger than 6 months with more extensive supplies of immunization products early in the season. Data from observational studies in future seasons may help to validate assumptions regarding the nature and duration of protection and refine scenario projections.

## Notes

Supplement 1.
eMethods.
**eFigure 1.** Time Series of RSV-Coded Hospitalizations in King County, Washington **eTable 1.** Reported Infant Immunizations in 2023-2024 and Population Denominators **eTable 2.** Model Parameters **eFigure 2.** Model Diagram **eFigure 3.** Sensitivity Analysis: Immunity Structures **eFigure 4.** Calibration of RSV Hospitalizations During the Pandemic and Postpandemic Periods **eFigure 5.** Starting Values for Immunization Effectiveness
eReferences.

Supplement 2.
Data Sharing Statement
