# Supplementary material for: Scenario Projections of Respiratory Syncytial Virus Hospitalizations Averted Due to New Immunizations
Source: JAMA Netw Open. 2025 Jun 11;8(6):e2514622. doi: 10.1001/jamanetworkopen.2025.14622 (PMC12159778; doi:10.1001/jamanetworkopen.2025.14622)
Supplement: Supplement 2. — Data Sharing Statement [file jamanetwopen-e2514622-s002.pdf]

Data Sharing Statement

Hansen. Scenario Projections of Respiratory Syncytial Virus Hospitalizations Averted Due to New Immunizations. *JAMA Netw Open*. Published June 11, 2025.
doi:10.1001/jamanetworkopen.2025.14622

Data
Data available: No

Additional Information
Explanation for why data not available: Raw data are not publicly available, however, contact information for researchers wishing to access the data is provided. Aggregated data and model code will be made publicly available for reproducibility.
